# Cell-Free Carbonic Anhydrase IX mRNA in Urine as Biomarker for Urogenital Cancers: The Relationship Between Urinary Extracellular RNA and Tumor-Cell-Associated RNA

**DOI:** 10.3390/cimb46120829

**Published:** 2024-12-06

**Authors:** Francesca Malentacchi, Irene Mancini, Donata Villari, Michael Forster, Andrea Marzocco, Ilaria Camilla Galli, Lorenzo Viola, Lorenzo Masieri, Gabriella Nesi, Pamela Pinzani

**Affiliations:** 1 Department of Experimental and Clinical Biomedical Sciences, University of Florence, Viale Gaetano Pieraccini 6, 51039 Florence, Italy; malentacchif@aou-careggi.toscana.it (F.M.); irene.mancini@unifi.it (I.M.); 2 Department of Minimally Invasive and Robotic Urologic Surgery and Kidney Transplantation, Careggi University Hospital, 50134 Florence, Italy; donata.villari@unifi.it (D.V.); andrea_marzocco@hotmail.it (A.M.); l.viola@unifi.it (L.V.); lorenzo.masieri@unifi.it (L.M.); 3 Institute of Clinical Molecular Biology, University Medical Center Schleswig-Holstein, 24105 Kiel, Germany; m.forster@ikmb.uni-kiel.de; 4 Histopathology and Molecular Diagnostics, Careggi University Hospital, 50134 Florence, Italy; galliic@aou-careggi.toscana.it; 5 Department of Health Sciences, University of Florence, Viale Gaetano Pieraccini 6, 50139 Florence, Italy; gabriella.nesi@unifi.it

**Keywords:** urinary-cell-free mRNA, urinary-tumor-cell-associated mRNA, CAIX, liquid biopsy, biomarkers in urine

## Abstract

Circulating tumor cells and cell-free nucleic acids are novel diagnostic, prognostic and predictive tools for non-invasive and cost-effective cancer detection in liquid biopsy. Carbonic anhydrase IX (CAIX) has been proposed as a biomarker in urogenital tumors and urine sediment. Our aim was to evaluate CAIX full-length percentage (CAIX FL%) in urine-cell-free RNA (cfRNA) and its relationship with tumor-cell-associated RNA (TC-RNA). CAIX FL% was quantified by reverse transcription quantitative polymerase chain reaction (RT-qPCR) in patients with prostate, kidney or bladder carcinoma. When cfRNA and TC-RNA were analyzed, CAIX FL% was significantly higher in urine samples from cancer patients than from controls. Using a 10% cutoff for CAIX FL%, specificity, sensitivity, positive and negative predictive values, as well as accuracy for TC-RNA were higher than for cfRNA in all urogenital cancers, but varied according to tumor type. CAIX FL% distribution in TC-RNA differed significantly (*p* < 0.001) between control and tumor samples (37.5% and 96.2%, respectively); similar results were obtained for each tumor type. Additionally, the 10% cutoff showed a 77.9% concordance between TC-RNA and cfRNA. In conclusion, urine is proposed as an alternative biofluid for investigating CAIX FL% in urogenital cancers, and this parameter can be reliably measured as cfRNA and TC-RNA with different predictive capabilities depending on tumor type.

## 1. Introduction

Circulating tumor cells (CTCs) and cell-free nucleic acids (cfNAs) are novel prognostic and predictive tumor biomarkers [1,2], and constitute the main targets in liquid biopsies (i.e., body fluids collected by non-invasive/minimally invasive procedures), allowing disease monitoring and tailoring of clinical treatment [3]. In particular, cfNAs have been identified as promising diagnostic tools for non-invasive and cost-effective cancer detection [4].

Urine is a suitable source of liquid biopsy for urogenital cancers and has been used in several studies for the detection of tumor-related biomarkers [5,6,7]. Tumor cells may exfoliate and be identified in urine sediment directly [8,9], or indirectly by analyzing tumor-related genetic alterations and mRNA tumor-specific signatures [10]. In oncologic patients, cfNAs isolated from blood, stool, and urine have proved valuable in monitoring therapeutic response and tumor relapse [3,11,12,13,14,15].

Cell-free RNA (cfRNA) is released from cancerous and non-cancerous cells. Despite the elevated levels of RNase in urine, cfRNA concentration remains high, probably on account of biopolymer complexes protecting it from nuclease degradation [16].

To date, a few studies have been published on the relationship between cf-NA in urinary supernatant and tumor-cell-associated NA (TC-NA) in urinary sediment (for a review see Ref. [6]) mostly focused on cfDNA and (among cfRNAs) on miRNAs in urine. Given the complexity of the topic and the impossibility of drawing definitive conclusions about the distribution of all the different biomarkers in urine, one can foresee the need to analyze each urinary biomarker to assess its clinical significance and positive aspects.

Carbonic Anhydrase IX (CAIX) is considered a biomarker in various malignancies for diagnostic and therapeutic purposes [17,18,19]. It is usually up-regulated in cancer [17,20,21], and linked to the development of tumor hypoxia, contributing to malignant progression and poor treatment outcome [18]. CAIX has been detected in body fluids such as serum and urine from patients affected by renal [21], bladder [22,23,24], breast [17], ovarian [25,26] and lung [20] cancer.

Moreover, we have previously demonstrated that CAIX full-length percentage (CAIX FL%) of the total CAIX mRNA can be identified in the urine sediment of patients with urological cancers and can serve as a non-invasive biomarker for prostate, bladder and kidney tumors [24,27].

In human cancers, carbonic anhydrase IX (CAIX) contributes to maintaining intracellular and extracellular pH under hypoxic conditions but also influences the regulation of cell proliferation and tumor progression. Two variants of CAIX mRNA have been reported: the full-length (FL) transcript, coding for the entire sequence deriving from the native CAIX gene with 11 exons, and a variant form without exons 7–8, which codes for a truncated protein lacking the transmembrane region, the intracellular tail and the catalytic domain [28]. This difference influences the cellular localization of the variants: whereas FL CAIX is typically a plasma membrane protein, AS has also a cytoplasmatic localization. In addition, AS seems to compete functionally with FL, by reducing the ability of FL to promote extra-cellular acidification. In conclusion, AS CAIX expression seems to be not related to the hypoxic adaptation of neoplastic cells during cancer progression and, to some extent, to act as a natural competitor of this mechanism of aggressive clone selection. Our previous results indicated that FL CAIX is the most accurate surrogate of hypoxic stress in cancer cells, representing the only variant with a prognostic role [27].

So far, little attention has been given to the levels of tumor-cell-free mRNA in urine, as most work on urinary mRNA levels has focused primarily on the use of whole urine or urine sediment.

Based on the most recent literature, urinary cfNAs are a promising, more sensitive alternative Liquid Biopsy approach than blood biopsies and urine sediments, particularly for clinical use in urogenital cancers [6].

Cell-free nucleic acids may reach the urine as a result of renal cfNA transport from the blood (transrenal origin) or directly from cells of the urogenital system in contact with this biological fluid (postrenal origin), mainly through apoptosis, necrosis, and active secretion. The biological function of urinary cfNAs is not yet completely understood, but DNA, RNA and small RNA are of interest for early detection of various oncological diseases.

Several advantages of urinary cfNA application may be mentioned. Compared with blood, this approach is definitely less invasive and the sample can be easily collected from patients without the help of specialised hospital staff. On the other hand, a pre-analytical blood procedure is undoubtedly simpler than for urine sediment, resembling that used for plasma. Extensive washing is not required to remove any impurities as in sediment, and the molecular target is less susceptible to contamination from normal cells and other elements that can be found in urine.

The aim of this paper is to evaluate urine as a liquid biopsy in urogenital cancers, focusing on the relationships between cfRNA and TC-RNA related to the CAIX mRNA target with special attention to its FL variant.

## 2. Materials and Methods

### 2.1. Cases and Controls

We recruited 52 patients with urogenital malignancies undergoing surgery at Careggi University Hospital, Florence, Italy. Pathological staging was performed in accordance with the 2017 AJCC TNM classification [29], and tumor grade was assessed following the 2022 WHO grading system [30]. The control group comprised 16 healthy subjects, matched for age and sex, and 20 patients with benign prostate hyperplasia (BPH).

### 2.2. Sample Collection

For each patient, a pre-surgical first void urine sample was collected, and 25 mL of fresh urine was centrifuged at 1500 rpm for 10 min at 4 °C. Urine sediments were washed twice with PBS before RNA extraction, and 2 mL of supernatant from the first urine centrifugation were collected in a separate sterile RNase-DNase-free tube for cfRNA isolation. The study was conducted in accordance with the guidelines of the Declaration of Helsinki and approved by the Local Ethical Committee (Prot. CEAVC 23090). Informed consent was obtained from all subjects involved in the study.

### 2.3. RNA Extraction, Quantity and Quality Check

RNA was extracted from the urine sediment and supernatant by means of an RNeasy Mini kit (Qiagen, Hilden, Germany) according to the manufacturer’s instructions and eluted with 30 µL of RNase-free water. Samples were treated with the RNase-free DNase set (Qiagen, Hilden, Germany) to eliminate DNA. For urine supernatant, 2 mL samples were treated following the protocol of the RNeasy Mini kit using 1200 μL RTL buffer with β-mercaptoethanol and adjusting the volumes of the subsequent buffers to the sample input volume. In the purification step on the column, the sample was uploaded several times until the entire volume was processed.

TC-RNA quantity and quality were assessed with a Nanodrop^®^ ND-1000 spectrophotometer (NanoDrop Technologies, Inc., Wilmington, DE, USA) and by capillary electrophoresis on a Lab-on-chip using an Agilent Bioanalyzer 2100 (Agilent Technologies, Palo Alto, CA, USA). Since the quantity of cfRNA cannot be measured with a spectrophotometer, 18S RNA expression was investigated by reverse transcription quantitative polymerase chain reaction (RT-qPCR). In particular, 8.2 µL of the eluate were reverse-transcribed with the TaqMan Reverse Transcription Reagent kit (Life Technologies, Carlsbad, CA, USA) in a 20 µL reaction mixture and 2.5 µL of cDNA were submitted to qPCR by adding 10.0 µL of PCR reaction mix containing 6.25 µL of TaqMan Universal Master Mix, 1x of Pre-Developed TaqMan Assay Reagents 18S (Life Technologies, Carlsbad, CA, USA) and 3.75 µL of water. Samples were incubated for 2 min at 50 °C, 10 min at 95 °C, and submitted to 40 amplification cycles at 95 °C for 15 s and 60 °C for 60 s in a 7900HT Fast Real-Time PCR System (Life Technologies, Carlsbad, CA, USA).

### 2.4. Quantification of CAIX mRNA

For CAIX measurement, 100 ng of TC-RNA or 8.2 µL of cfRNA were reverse-transcribed with the TaqMan Reverse Transcription Reagent kit (Life Technologies, Carlsbad, CA, USA). Subsequently, 2.5 µL of cDNA, from cfRNA or TC-RNA, were amplified by qPCR for total and FL CAIX in a duplex assay format, in a reaction mix containing 6.25 µL of TaqMan Universal Master Mix, 300 nM of forward and reverse primers for each set, and 200 nM of each fluorescent probe. Primer and probe sequences have previously been reported [24]. Thermal cycling conditions were 2 min at 50 °C, 10 min at 95 °C followed by 40 cycles of amplification at 95 °C for 15 s and 60 °C for 60 s in ABI Prism 7900 Sequence Detector PE (Life Technologies, USA). Quantification of targets was achieved using an external reference curve obtained by serial dilutions of a plasmid containing FL CAIX (pGEX-3X-CA9) [24]. Results for CAIX mRNAs were expressed as copies/mL and the percentage of FL (CAIX FL%) was calculated with respect to the total amount of CAIX mRNA.

### 2.5. Validation Stategies

The statistical validation of the proposed 10% cut-off for CAIX FL% was performed on an external cohort consisting of 81 bladder cancer patients (urothelial carcinomas) and a group of 93 age and gender-matched subjects with no urologic malignancy history, already acting as a normal control in a publication by our group [24]. The proposed cut-off was calculated on the basis of Receiver Operating Characteristic (ROC) curves generated to compare the diagnostic performances of total CAIX and FL isoform expressed as CAIX FL% in urine sediment. The maximum potential effectiveness of these biomarkers was calculated by the Youden Index to express the cut-off point that optimizes biomarker differentiating ability when equal weight is given to sensitivity and specificity. The proposed 10% cut-off corresponds to a clinical sensitivity of 0.93 and clinical specificity of 0.76 [24].

### 2.6. Statistical Analysis

Statistical analysis was carried out using the SPSS software package version 29.0.2.0 (20) (SPSS Inc., Chicago, IL, USA). A simple descriptive analysis was performed using frequencies for categorical variables and median values for continuous variables. The comparison between groups was assessed by appropriate tests (chi-square test for categorical variables, and Mann–Whitney test or Kruskal–Wallis test for continuous variables, for two or more groups, respectively). Correlation coefficients between different parameters were calculated by appropriate tests. A ROC (Receiver Operating Characteristics) analysis including the area under the curve (AUC) was performed to define the diagnostic performance of specific biomarker. The Youden Index (defined as Y = sensitivity + specificity − 1) was used selecting the optimal threshold value (cut-off point) for the markers under study. Statistical analysis was accomplished using the SPSS software package (SPSS Inc., Chicago, IL, USA). In all tests, *p* ≤ 0.05 was considered statistically significant.

## 3. Results

### 3.1. Cohort Characteristics

The study population consisted of 52 patients affected by urogenital tumors (10 with prostatic acinar adenocarcinoma, 7 with renal cell carcinoma, and 35 with urothelial bladder cancer) and 20 BPH patients. In addition, 16 healthy subjects have been included in the study as the control group. Patient clinicopathological features are detailed in Table 1.

### 3.2. Urinary CAIX mRNA

#### 3.2.1. Cell-Free RNA

To verify the possibility of detecting CAIX mRNA in the supernatant, we performed a pilot study on urine samples from five bladder cancer patients and five controls. Median (range) levels of total and FL mRNA were as follows: total CAIX was 66 (2.5–235) copies/mL in bladder cancer patients vs. 810 (130–1130) copies/mL in controls; FL CAIX was 22 (0.6–235) copies/mL in bladder cancer patients vs. 3 (2.4–7) copies/mL in controls.

**Figure 1:**
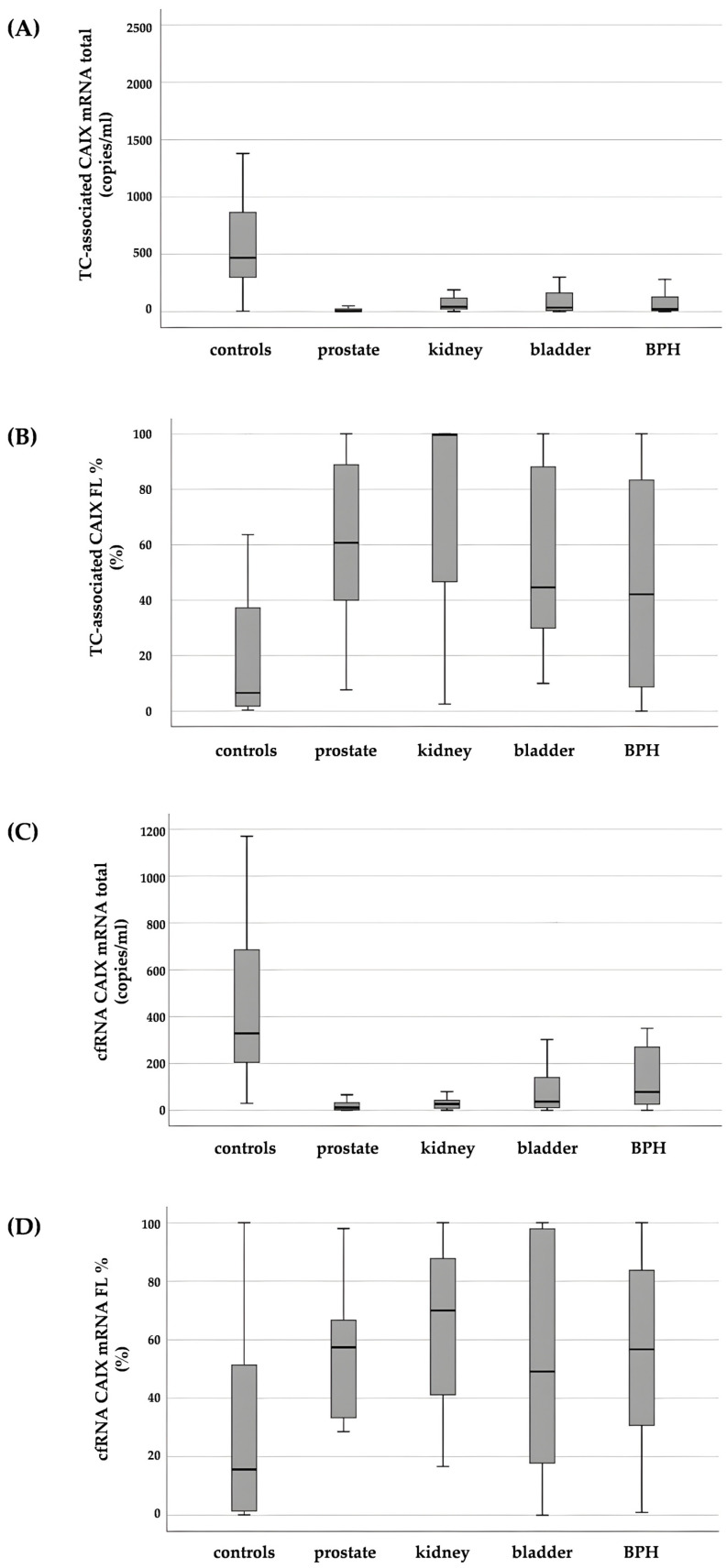
Total CAIX mRNA in urine sediment (**A**) and supernatant (**C**), CAIX FL% in urine sediment (**B**) and supernatant (**D**).

All supernatant samples in our series were positive for 18S, total and FL CAIX. Total CAIX was significantly higher in healthy subjects than in patients with urogenital cancer (*p* < 0.001), regardless of tumor type (prostate, kidney, bladder), or BPH (*p* < 0.001) (Figure 1C). Moreover, total CAIX was greater in BPH patients than in those with prostate cancer (*p* = 0.003), while CAIX cfRNA levels were highest in the bladder and lowest in prostate carcinoma (Table 2).

As previously demonstrated, total CAIX is not the best parameter to be used as a tumor biomarker since it includes also the splicing variant AS that is not related to the hypoxic stress in cancer cells [24]. Therefore, we tested for the presence of FL CAIX, which was detectable in all tumors with the highest levels in the prostate and the lowest in the kidney, although differences were not significant. As a normalizing factor and in order to be able to compare the data obtained for different types of urogenital cancers, we expressed the FL results as the percentage of the total CAIX value (Table 2) and found to be significantly higher in cancer patients than in controls (overall: *p* = 0.014; prostate: *p* = 0.025; kidney: *p* = 0.035; bladder: *p* = 0.050) and in BPH patients than in healthy subjects (*p* = 0.013) (Figure 1D).

#### 3.2.2. Tumor-Cell-Associated RNA

Total CAIX was detectable in all sediment samples (Table 2) and, as expected, was higher in controls than in urogenital cancers (*p* < 0.001 with distribution varying for each tumor type (prostate, *p* < 0.001; kidney, *p* = 0.004; bladder, *p* = 0.001). Values differed significantly between BPH and prostate cancer patients (*p* = 0.002, Figure 1A).

FL CAIX was detectable in all tumors, with the highest levels in the bladder and the lowest in the prostate. CAIX FL% was lower in controls than in urogenital cancers when taken together (*p* < 0.001) (Table 2) and split on the basis of tumor primary site (prostate *p* = 0.005; kidney *p* = 0.021; bladder *p* = 0.001) Furthermore, CAIX FL% was lower in BPH than in prostate cancer patients (*p* = 0.010, Figure 1).

### 3.3. Comparison of CAIX Expression Between Cell-Free and Tumor-Cell-Associated RNA

Although Total, FL and FL% show similar distribution in sediment and supernatants when comparing all tumors with healthy subjects, suggesting the same behavior in the two compartments, their analysis within each individual tumor type reveals a different relationship between the two districts. In fact, in Table 2, it can be seen that in BPH, prostate and bladder tumors, the total CAIX amount in cfRNA is higher than the corresponding TC-RNA, while the opposite is true in the kidney.

A linear regression analysis was performed between cfRNA and TC-associated mRNA values, and the resulting R-values, reported in Table 3, show a very high correlation between total CAIX mRNA in the supernatant and sediment in all tumors under study, while the correlation is lower in benign pathology (BPH) and even more so in the physiological conditions of healthy subjects.

The strong correlation between cfRNA and TC-RNA is maintained for the FL isoform in the prostate, BPH and kidney, while bladder tumors are closer to controls. This variability in behavior between CAIX tot and FL is reflected in the low correlation coefficients between the two compartments found for FL%.

Based on this consideration, the distribution and origin of CAIX tot and FL may vary in the supernatant and sediment depending on tumor type, resulting in different panoramas depending on the sample used for measurement.

On the other hand, when evaluating our samples on the basis of a previously established 10% cutoff for positivity, we found a general agreement between the number of CAIX FL% positive samples in cfRNA and TC-RNA with percentages of 80.8% (55/68) and 82.3% (56/68), respectively. Discrepant results accounted for 22% of all samples (chi-square, *p* = 0.029) (Table 4).

### 3.4. Clinical Relevance of Urinary CAIX mRNA

**Figure 2:**
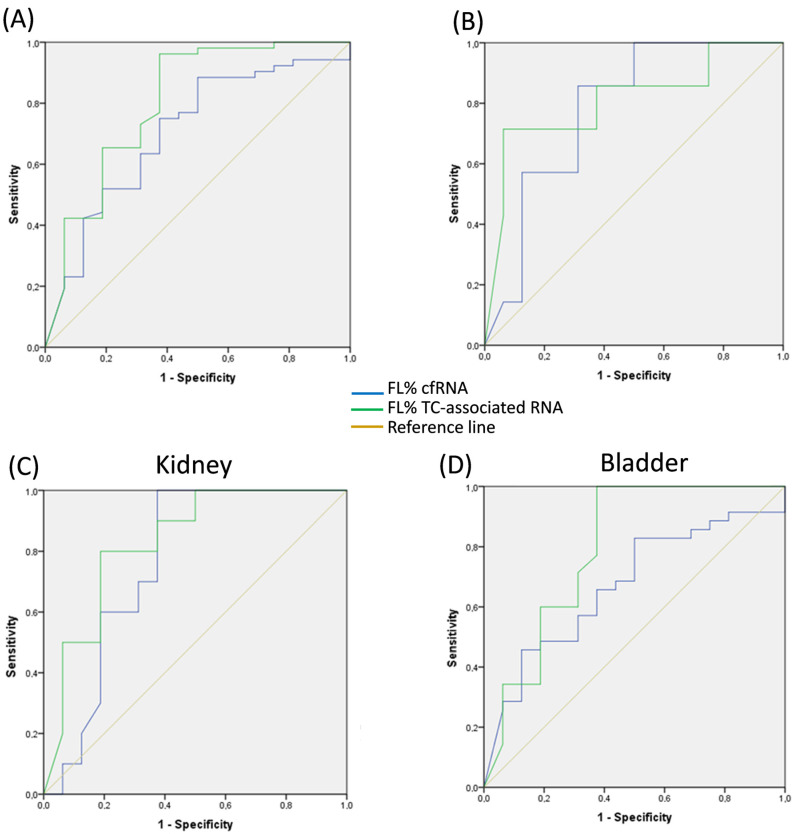
ROC curves univariate logistic analysis. Comparison of the predictive value of CAIX FL% cfRNA and TC-RNA from urine samples of patients with urogenital cancers: (**A**) overall evaluation in all types of cancer; (**B**) prostate, (**C**) kidney and (**D**) bladder cancer.

We investigated the predictive capability (i.e., diagnostic performance) of CAIX FL% in cfRNA and TC-RNA by ROC curve analysis (Figure 2), with significant results (*p* = 0.014 and *p* = 0.001, respectively) and area under the curve (AUC) values of 0.704 and 0.803, respectively. This trend was maintained for each individual tumor.

Applying the 10% cutoff, TC-RNA showed 62.5% specificity, 96.1% sensitivity, 89.3% positive predictive value (PPV), 83.3% negative predictive value (NPV), and 88.2% accuracy. A similar trend was observed for cfRNA, although its corresponding parameters were lower. These values differed slightly when individual tumor types were analyzed (Table 5). Of note, sensitivity was high in all tumor types, while NPV reached 100% for cfRNA in the prostate and kidney, and for TC-RNA in the bladder. CAIX FL% distribution in TC-RNA was significantly different (chi-square, *p* < 0.001) between controls (37.5%, >10% cutoff) and urogenital tumors (96.2%, >10% cutoff) as well as among cancer types (bladder: 100.0%, *p* < 0.001; kidney: 85.7%, *p* = 0.033; prostate: 90.0%, *p* = 0.008). Similar data were obtained for cfRNA, with a significant difference (chi-square, *p* = 0.009) between controls (56.3%, >10% cutoff) and urogenital tumors (88.5%, >10% cutoff), and between controls and individual cancer types (bladder: 82.9%, *p* = 0.043; kidney: 100.0%, *p* = 0.036; prostate: 100.0%, *p* = 0.014).

## 4. Discussion

Nucleic acids in body fluids have been extensively investigated for their role as biomarkers for diagnostic, prognostic and predictive purposes in cancer [1,2,3,4,5,12,19,20,25,31,32], including urogenital malignancies [11,21,24,32,33]. The aim of this study was to assess CAIX mRNA as a cancer-derived molecular biomarker in prostate, kidney and bladder carcinomas, analyzing its presence in cfRNA in relation to paired TC-RNA urine samples. This aspect, to our knowledge, has not been previously studied regarding CAIX in urogenital cancers and may open up new avenues of research.

Increased CAIX expression is associated with an aggressive phenotype and predicts poor outcomes in breast, cervical, non-small cell lung, esophageal, and gastric cancer [18,20,22,25,26,28,31,32,34]. In previous studies, we showed significantly higher CAIX FL% levels in urogenital cancer patients than in healthy subjects, both in tumor tissues and corresponding sediments [27]. The use of urine as a surrogate for tumor tissue proved to be feasible, and the 10% cutoff of CAIX FL% was effective in stratifying patients [24].

Herein, we have further demonstrated that overall all the tumors under investigation exhibit significantly higher CAIX FL% compared to controls, with overall slightly greater values in TC-RNA than cfRNA. Since the urine contains a lot of nucleases, including RNases (their diversity is described in ref. [35]), a higher stability of mRNA in intact cells compared to cfRNA can be hypothesized to explain this finding. In fact, the use of urinary mRNA for the development of diagnostic systems for various diseases remains quite a challenge, since a high concentration of enzymes that hydrolyze RNA complicates the processing of cell-free RNA, including the isolation stage [35].

We have shown that the distribution and origin of CAIX tot and FL may vary in the supernatant and sediment depending on the type of tumor, being distributed differently between the cellular compartment and the supernatant. These discrepancies may be due to both plasma-transrenal and postrenal origin of the cfRNA [36] and variability in the levels of mRNA released in urine due to the possible difference between the amounts resulting from apoptosis, necrosis, and those resulting from active secretion, i.e., as part of vesicles.

To appraise the clinical significance of CAIX FL%, we applied a 10% cutoff and showed that CAIX FL% measured in urine sediment enables to accurate detection of urogenital cancers. We also evaluated the predictive capability of this parameter for TC-RNA and observed an overall sensitivity of 96.1% and specificity of 62.5%. A similar trend was seen for cfRNA, although specificity was lower (43.8%) and sensitivity higher (up to 100%) for prostate and kidney cancers. Of note, NPVs ranged between 83.3% and 100% except for bladder carcinoma (53.9%).

Our results contribute to the emerging field of urine-based liquid biopsy for urogenital malignancies. Current methods for diagnosing and monitoring these tumors are invasive and not sufficiently sensitive or specific, thus effective body-fluid biomarkers are strongly needed [37].

Urine is an optimal source of tumor-derived material since it is easy to obtain with no discomfort for the patient. In addition to the possibility of collecting the sample at home, thus allowing more frequent testing for close monitoring of the patient, this approach can also facilitate the detection of early-stage cancers [6]. However, only a few mRNA-based urine liquid biopsy approaches have been employed so far [36].

The major advantages of the test under investigation include its simplicity, low cost and high clinical sensitivity. Liquid biopsy analysis focusing on cfRNA in the urine supernatant, which is purer than the urine pellet often contaminated with normal cells, immune cells, debris and bacteria, has the enormous advantage of reducing all those interference factors that are the main limitation to the use of urine in most highly sensitive tests. [36].

Urinary tests based on the detection of cell-free nucleic acids might be considered an attractive and practical alternative to the analysis of urine sediment and blood liquid biopsies for urogenital cancers [6]. However, the relatively low clinical specificity seen in our patient cohort is a limitation of the proposed biomarker that need to be further investigated in larger cohort studies.

The recent trend in the field of tumor biomarkers (and more specifically regarding Liquid Biopsy) proposes a multimarker/multimodal testing procedure to improve the clinical performances of molecular biology tests. Further studies are needed to explore the feasibility of associating a routine tumor-specific biomarker with the CAIX FL% parameter to improve the specificity of the test.

## 5. Conclusions

Identification of tumor-related biomarkers in urogenital malignancies is challenging due to the heterogeneity of the tissues of origin (prostate, bladder, kidney) and the invasive biopsy procedures employed for disease monitoring. Urine proves to be useful in detecting biomolecules for diagnostic, prognostic and therapeutic purposes, regardless of tumor type. Assessment of cfRNA can be conducted in this biofluid, and CAIX FL% cfRNA seems a promising and informative biomarker to enhance early diagnosis of urogenital cancers. Larger prospective studies are, however, mandatory to fully validate its robustness and clinical utility. Indeed, we can expect a broadening of the landscape for possible applications of this test based on recent advances in CAIX biology and pharmacology in solid tumors other than urogenital cancers [38].

## Figures and Tables

**Table 1:** Patient clinicopathological characteristics.

	Prostate Cancer n = 10	Renal Cancer n = 7	Bladder Cancer n = 35
Age (mean, range)	68, 47–77	67, 52–82	72, 48–92
Sex		M (1, 14%)	n = 26 (74%)
		F (6, 86%)	n = 9 (26%)
Histology	Acinar (10, 100%)	Chromophobe (3, 43%) Clear cell (4, 57%)	Papillary urothelial carcinoma (27, 77%) Invasive urothelial carcinoma (8, 23%)
Tumor grade	GG1 (3, 30%) °	G1 (2, 50%) *	LG papillary (12, 44%)
	GG2 (2, 20%)	G2 (1, 25%)	HG papillary (15, 56%)
	GG3 (2, 20%)	G3 (1, 25%)	
	GG4 (2, 20%)		
	GG5 (1, 10%)		
Tumor stage	T1a (1, 10%)	pT1a (3, 43%)	pTa (14, 40%)
	pT2 (5, 50%)	pT1b (2, 29%)	pT1 (14, 40%)
	pT3a (2, 20%)	pT2a (1, 14%)	pT2a (2, 6%)
	pT3b (2, 20%)	pT3a (1, 14%)	pT2b (1, 2%)
			pT3a (2, 6%)
			pT4a (2, 6%)

° WHO Classification of Tumours grade (WHO grade). * WHO/International Society of Urological Pathology (ISUP) grading system for clear renal cell carcinoma. GG, grade group; LG, low-grade; HG, high-grade.

**Table 2:** Total CAIX and CAIX FL% (cfRNA and TC-RNA) in controls, urogenital tumors taken together and split on the basis of tumor primary site.

		Total CAIX (Copies/mL) Median (Range)		CAIX FL% Median % (Range)	
	n	cfRNA	TC-RNA	cfRNA	TC-RNA
**Controls**	16	270 (10–1170)	470 (5–2110)	18.5 (0.1–100)	6.6 (0.4–100)
**BPH**	20	80 (0–350)	23 (1.2–308)	45.5 (1.4–99)	64.8 (0–100)
**All tumors**	52	25 (0–760)	25 (0.3–580)	41.20 (0–100)	46.49 (0–100)
**Prostate**	10	13.5 (0.6–287)	3.8 (0.7–49.6)	31.7 (0–80)	48.3 (0–100)
**Kidney**	7	27 (0.5–190)	40.6 (0.4–190)	40.7 (0–100)	99.8 (0–100)
**Bladder**	35	32.4 (0–760)	31.5 (0.3–580)	49.1 (0–100)	42.9 (0–100)

**Table 3:** R values from linear correlation between cell-free and tumor-cell-associated RNA.

	CAIX mRNA Total	CAIX mRNA FL	CAIX FL%
All samples	0.486	0.659	0.358
BPH	0.895	0.917	0.895
Controls	0.545	0.642	0.362
All tumors	0.922	0.642	0.239
Prostate	0.906	0.968	0.307
Kidney	0.915	0.876	0.534
Bladder	0.923	0.557	0.103

**Table 4:** Correlation between CAIX FL% positive and negative samples by analysis of tumor-cell-associated RNA (TC-RNA) in the urine sediment and cell-free RNA (cfRNA) in urine supernatant. Positivity was estimated assuming the cut-off of 10% (Chi-square, *p* < 0.05).

	CAIX FL% TC-RNA			
CAIX FL% cfRNA		Positive (>10%)	Negative (<10%)	Total
	Positive (>10%)	48	7	55
	Negative (<10%)	8	5	13
	total	56	12	68

**Table 5:** Sensitivity, specificity, positive and negative predictive values, and accuracy of CAIX FL% applying the 10% cutoff for all urogenital tumors and individual tumor types.

Parameters (%)						
		Sensitivity	Specificity	Positive Predictive Value	Negative Predictive Value	Accuracy
All tumors	*TC-RNA*	96.1	62.5	89.3	83.3	88.2
	*cfRNA*	88.5	43.8	83.6	53.9	77.9
Prostate	*TC-RNA*	90.0	62.5	60.0	90.9	73.0
	*cfRNA*	100.0	43.8	52.6	100.0	65.3
Kidney	*TC-RNA*	85.7	62.5	50.0	90.9	69.6
	*cfRNA*	100.0	43.8	43.8	100.0	60.9
Bladder	*TC-RNA*	100.0	62.5	85.4	100.0	88.2
	*cfRNA*	82.9	43.8	76.3	53.9	70.6

## Data Availability

The raw data supporting the conclusions of this article will be made available by the authors upon reasoned request.
